# Phase Ib study of CP-868,596, a PDGFR inhibitor, combined with docetaxel with or without axitinib, a VEGFR inhibitor

**DOI:** 10.1038/sj.bjc.6605941

**Published:** 2010-10-19

**Authors:** M Michael, G Vlahovic, K Khamly, K J Pierce, F Guo, A J Olszanski

**Affiliations:** 1 Peter MacCallum Cancer Centre, Consultant Medical Oncologist, Division of Haematology and Medical Oncology, Chair of GI Clinical Service, Locked Bag 1, A’Beckett Street, Victoria 8006, Australia; 2 Duke University Medical Center 3335, Durham, NC 27710, USA; 3 Peter MacCallum Cancer Centre, Locked Bag 1, A’Beckett Street, Victoria 8006, Australia; 4 Pfizer Oncology, 50 Pequot Avenue, MS 6025-A3275, New London, CT 06320, USA

**Keywords:** CP-868,596, docetaxel, axitinib, phase Ib, advanced solid tumours

## Abstract

**Background:**

Tumoural interstitial hypertension, possibly modulated by platelet-derived and vascular endothelial growth factor receptors (PDGFR and VEGFR), may mediate resistance to chemotherapy.

**Methods:**

Forty-eight patients with advanced solid tumours received oral PDGFR inhibitor CP-868,596 (60–100 mg twice daily (BID)) and docetaxel (75–100 mg m^–2^), or CP-868,596 (60 mg BID), docetaxel (75 mg m^–2^), and VEGFR inhibitor axitinib (5 mg BID).

**Results:**

The CP-868,596/docetaxel was escalated as above. The CP-868,596/docetaxel/axitinib was not dose escalated because of increased incidence of mucositis-like adverse events (AEs) with concurrent neutropenia relative to that expected for docetaxel. All tested regimens were tolerable, including 100 mg BID CP-868,596 (recommended phase II dose) plus 100 mg m^–2^ docetaxel (maximum approved dose). Most treatment-emergent AEs were mild–moderate and reversible, commonly including nausea, diarrhoea, vomiting, constipation, fatigue, and anaemia (CP-868,596/docetaxel), and hypertension, lethargy, diarrhoea, and fatigue (CP-868,596/docetaxel/axitnib). Pharmacokinetics were unaffected by co-administration. Twenty-one patients achieved stable disease, including all seven evaluable on CP-868,596/docetaxel/axitinib. All nine CP-868,596/docetaxel/axitinib patients received therapy for a median of six (range, 3–16) cycles.

**Conclusions:**

The CP-868,596/docetaxel was well tolerated, but increased efficacy was not observed. Addition of axitinib delivered greater benefits than expected in the number of patients achieving prolonged stable disease with a moderate increase in AEs.

Progression of tumours requires angiogenesis, driven by vascular endothelial growth factor (VEGF), to form new capillary networks. Subsequently, platelet-derived growth factor (PDGF) activation of the tyrosine kinase PDGF receptor-*β* (PDGFR-*β*) is responsible for the migration of supporting pericytes to stabilise this network (Hellstrom *et al*, 1999; Saharinen and Alitalo, 2003) and also for increased endothelial cell VEGF expression (Hellstrom *et al*, 1999; Wang *et al*, 1999; Pietras *et al*, 2003a).

Tumoural blood vessels have variable diameters, sparse pericyte coverage, and are leaky to solutes (Saharinen and Alitalo, 2003). In addition, excessive PDGF production within tumours stimulates stromal cells via collagen-binding integrin-*α*_2_*β*_1_ (Reed *et al*, 1997; Pietras *et al*, 2001; Ostman and Heldin, 2007). Overall, these factors contribute to an increase of the interstitial fluid pressure (IFP) within tumours, resulting in tumoural interstitial hypertension (TIH). The TIH reduces the hydrostatic gradient from capillary to tissues and decreases the diffusion of molecules, including drugs, into tumours (Gullino *et al*, 1964; Jain, 1987), with therapeutic consequences.

The PDGFR-*β* is commonly overexpressed in the stroma and pericytes of several solid organ tumour types (Reed *et al*, 1997; Ostman and Heldin, 2007). Preclinical data have shown that PDGFR inhibition reduces tumoural IFP and increases the intracellular concentrations of cytotoxics (Pietras *et al*, 2001, 2003b; Vlahovic *et al*, 2006). The VEGF inhibition has also been shown to reduce tumour IFP (Willett *et al*, 2005). Thus, concurrent modulation of both angiogenesis and TIH may achieve improved tumoural drug exposure and increase therapeutic efficacy. Nevertheless, the potential of simultaneously inhibiting these targets has not been assessed clinically.

The CP-868,596 is a potent, oral, selective inhibitor of PDGFR-*β* tyrosine kinase, with an IC_50_ of 0.4 ng ml^–1^ and is >50–100-fold more selective for PDGFR *vs* other related kinases (Lewis *et al*, 2009). The first-in-human phase 1 study of single-agent CP-868,596 identified a recommended dosage of 100 mg twice daily (BID) with food (Lewis *et al*, 2009). Dose-limiting toxicities (DLTs) identified included haematuria, transaminitis, insomnia, and nausea/vomiting. The majority of adverse events (AEs) were of mild or moderate severity, with nausea and vomiting – which were mitigated by administration with food – most commonly reported. Studies in xenograft models have suggested that the cytotoxicity of docetaxel and gemcitabine are increased by combination with CP-868,596 (Whalen *et al*, 2005). In order to understand the clinical effects of combining PDGFR inhibition with chemotherapy, this phase Ib trial evaluated the safety and efficacy of the combination of CP-868,596 and docetaxel. Also explored was the effect of concurrently inhibiting PDGFR and VEGF receptors (VEGFRs) by adding axitinib (AG-013736), a potent and selective oral inhibitor of VEGFR-1, -2, and -3, to the combination.

## Patients and methods

### Patient selection

Eligible patients had the following: (1) histologically/cytologically proven solid organ tumours refractory/resistant to therapy; (2) ECOG performance status 0–2; (3) adequate organ function – absolute neutrophil count ⩾1.5 × 10^9^ per l, platelets ⩾100 × 10^9^ per l, haemoglobin ⩾8.5 g per 100 ml, serum creatinine ⩽1.5 × upper limit of normal (ULN), total serum bilirubin ⩽ULN, aspartate aminotransferase/alanine aminotransferase ⩽1.5 × ULN, urine protein <1+ on urinalysis or 24-h urine protein <500 mg if initial urine protein ⩾1+, QTc ⩽500 ms; and (4) for patients receiving axitinib – baseline blood pressure <140/90 mm Hg.

Patients were excluded for the following: (1) history of grade ⩾2 haemoptysis or grade 1 haemoptysis within 30 days of study entry; (2) surgery within 4 weeks of entry; (3) prior systemic therapy within 3 weeks of entry; (4) anticoagulant therapy; (5) use of cytochrome CYP3A4 or P-glycoprotein inhibitors within 1 week, or inducers within 3 weeks of entry; (6) cardiac history including prolonged QTc interval or deep-vein thrombosis within 3 months of entry; and (7) for patients receiving axitinib – central lung lesions involving major vessels and previous therapy with antiangiogenesis agents.

### Study design

This was a multicenter dose-escalation phase Ib study of CP-868,596 plus docetaxel +/− axitinib. Study design is available online (Supplementary Figure 1). The primary objective was to define the maximum tolerated dose of the double and triple combinations. However, should dose levels (DLs) combining the recommended phase 2 doses of investigational agents (CP-868,596 or axitinib) with approved doses of docetaxel be well tolerated, dose escalation would stop at this point and the relevant DL(s) expanded for further pharmacodynamic and efficacy studies.

The primary end point was DLTs occurring on combination therapy during the first-treatment cycle, assessed using the National Cancer Institute Common Terminology Criteria for Adverse Events (NCI CTCAE), version 3.0. The DLTs were defined as (1) grade 4 neutropenia for >7 days; (2) febrile neutropenia requiring intravenous (IV) antibiotics; (3) grade 4 thrombocytopenia for >7 days or with bleeding requiring transfusion; (4) grade ⩾3 clinically significant non-haematologic toxicity for >7 days despite appropriate treatment; and (5) inability to begin cycle 2 within 2 weeks of scheduled dosing because of persistent >grade 1 toxicity.

Dose escalation was allowed if none of the three or one of six patients experienced a DLT on combination therapy during cycle 1. The DLs could be expanded based on adequate safety/tolerability and provision was made for dose de-escalation in the event of DLTs in ⩾2 of three or six patients.

The MTD was defined as the highest DL at which <33% of patients experienced a DLT within days 22–42 of cycle 1 (DLs 1–3) or days 1–28 of cycle 1 (DL 4). Sample size was determined empirically depending on safety/tolerability.

The study was performed in accordance with International Conference on Harmonization Good Clinical Practice guidelines, and institutional ethics committee approval was obtained. All participants provided written consent.

### Treatment

Patients were enrolled into four dose-escalation DLs of 3–6 patients. Patients enrolled into DLs 1–3 received CP-868,596 orally BID plus docetaxel once every 21 days (starting day 22 of cycle 1). Patients enrolled into DL 4 received CP-868,596 BID plus axitinib 5 mg BID plus docetaxel once every 21 days (starting day 8 of cycle 1).

Each cohort included a lead-in phase. In DLs 1–3, the lead-in phase was 21 days, such that cycle 1 was 42 days. The lead-in phase allowed for CP-868,596 steady-state pharmacokinetics and pharmacodynamic studies (biomarkers and dynamic contrast-enhanced magnetic resonance imaging (DCE-MRI) tumoural analysis). During the lead-in phase, patients received CP-868,596 100 mg BID on an empty stomach over 14 days with a 5-HT_3_ antagonist, followed by a cohort dose of CP-868,596 BID (60 or 100 mg), given with food.

In DL 4, the lead-in phase was 7 days, such that cycle 1 was 28 days. The lead-in phase allowed for CP-868,596 and axitinib to potentially ‘normalise’ tumour vasculature (Jain, 2005). During the lead-in phase, patients received CP-868,596 60 mg BID plus axitinib (5 mg BID), given with food.

For all cohorts, docetaxel (75 or 100 mg m^–2^ IV over 1 h) was administered after the lead-in phases and then every 21 days. Premedication was standard.

### Evaluation of safety and tolerability

All patients who received at least one dose of study medication (CP-868,596 or axitinib) were evaluable for safety. Safety data were collected prospectively. The AEs were graded according to NCI CTCAE, version 3.0. The ECGs were performed at baseline and 3 h post-docetaxel therapy in cycle 1. Symptomatic QTc prolongation required discontinuation from the study. The safety evaluation period extended from the last dose of study drug through 28 days’ follow-up.

### Antitumour activity

All patients who received study treatment and had measurable disease at baseline were evaluable for response using response evaluation criteria in solid tumours (Therasse *et al*, 2000). Radiological assessments were conducted at screening and within 7 days prior to cycle 1 docetaxel dose for DLs 1–3, and at screening and cycle 2 day 1 for DL 4. Subsequent assessments were every other cycle for all DLs.

### Pharmacokinetic methods and analyses

In DLs 1–3, blood samples for determination of trough CP-868,596 serum concentrations were taken from all patients predose (0 h) on day 14. For the MTD expansion cohort (DL 2), blood samples were collected for the pharmacokinetic profiling of CP-868,596 alone and for the combination CP-868,596 plus docetaxel: samples were taken during cycle 1 at 0 h (predose) and 0.5, 1, 1.25, 1.75, 3, 6.5, and 12 h post-dose on days 21 (CP-868,596) and 22 (CP-868,596 and docetaxel). For CP-868,596 and docetaxel, 24- and 48-h post-dose samples were collected on days 23 and 24, respectively.

In DL 4, sampling was conducted on day 8 (CP-868,596, axitinib, and docetaxel) and day 9 (CP-868,596 and axitinib) of cycle 1: samples were collected at 0 h (predose) and 1, 1.75, 3, 4.5, and 6.5 h post-dose on day 8 and at 0 h (predose) on day 9 for CP-868,596 and axitinib. Docetaxel concentrations were assessed at 1 (end of infusion), 1.75, and 6.5 h post-dose on day 8.

Concentrations of CP-868,596, docetaxel, and axitinib were determined using validated HPLC/tandem mass spectrometry (HPLC/TMS) methods. Analytes were detected using an Applied-Biosystem Sciex API 3000/4000 LC/TMS system (MDI SCIEX, Concord, ON, Canada) operating in positive ion Electrospray mode. Non-compartmental pharmacokinetic analysis was performed using WinNonlin, version 3.2 (Pharsight, Mountain View, CA, USA). Parameters estimated for CP-868,596 and axitinib included maximum observed plasma concentration (*C*_max_), time to *C*_max_ (*T*_max_), and area under the plasma concentration–time curve from time zero to the dosing interval (AUC_0–tau_), and for docetaxel: *T*_max_, half-life (*t*_1/2_), AUC_0–48_, and AUC_0–infinity_.

### Pharmacodynamic assessments

The DCE-MRI was performed between days –3 and 0, and days 10 and 14, in a subset of patients in DL 2. A 3-T unit was used with a torso-phased array for imaging and a three-dimensional fast spoiled gradient-recalled echo sequence to acquire T1-weighted images before, during, and after IV administration of 0.1 mmol kg^–1^ Magnevist, infused at 3 ml s^–1^. The DCE time course in the tumour was analysed on a pixel-by-pixel basis by fitting the data to the Kety equation and by integrating the area under the initial (i.e. the first 90 s) uptake curve. The contrast-agent concentration time course in plasma and tissue was inferred from the change in T1-weighted signal as a function of time. The Kety equation fitted the tissue contrast-agent time course to a two-compartment model: d*C*_t_/d*t*=*K*^trans^(*C*_p_(*t*)–[*C*_t_(*t*)/*V*_e_]). *C*_p_ and *C*_t_ are plasma and tissue concentration, respectively, *K*^trans^ is volume endothelial transfer constant between plasma and the extracellular extravascular space (EES), and *V*_e_ is fractional EES volume (Ashton *et al*, 2008).

Blood samples for the evaluation of biomarkers potentially related to the PDGFR signalling pathway (serum Src homology phosphatase (SHP)2, phopho(p)-SHP2, pAKT, and pS6) were collected within 4 days of baseline and on day 14 in each cohort. Protein levels were analysed using gel electrophoresis (SDS–PAGE) and the LI-COR immunodetection system with appropriate antibodies.

### Statistical analyses

Sample size was selected empirically: 36–60 patients were considered required to determine the MTD. Descriptive statistics were used when analysing patient demographics, safety, and tumour response data. Paired *t*-test was used to assess the impact of co-administration of docetaxel on CP-868,596 pharmacokinetics.

## Results

### Patient characteristics

Forty-eight patients received at least one dose of study medication. Patient demographics are summarised in Table 1. The majority of patients were male, with non-small cell lung cancer (20.8%) and prostate cancer (12.5%) predominating. Approximately 30% had received at least three prior therapy regimens. Only four patients had been treated with docetaxel prior to enrolling into the study.

### Drug exposure, DLTs, and expansion DL

DLs tested, DLTs, and median duration of therapy are presented in Table 2. The median duration on therapy at DLs 1–4 ranged from 1.48 to 5.3 months, the maximum being 12.47 months.

The DLTs for CP-868,596 plus docetaxel were febrile neutropenia, nausea, and vomiting. The DLT for CP-868,596 plus axitinib and docetaxel was infection associated with febrile neutropenia. The DL 2 was chosen for expansion as no DLT was observed in the initial dose-escalation cohort of six patients and this DL combined the recommended phase 2 dose of CP-868,596 with the most commonly used dose of docetaxel.

Secondary to an increased incidence of mucositis-like AEs (mucosal inflammation, oral pain, and/or stomatitis) and neutropenia (based on laboratory values and reported AEs), relative to that expected for docetaxel alone, the planned escalation of the triplet to CP-868,596 100 mg BID/docetaxel 75 mg m^–2^/axitinib 5 mg BID was not attempted. All nine patients in DL 4 had grade ⩾3 neutropenia (67% grade 4), five had concurrent mucositis-like AEs, and three had neutropenic fever (33%).

A similar pattern was seen in the doublet cohorts (DLs 1–3), in which the incidence of neutropenia and mucositis-like AEs was high. Of 32 patients who received both CP-868,596 and docetaxel, 94% (30 patients) had grade 3/4 neutropenia and 43% (13 out of 30) mucositis-like AEs. Five patients, all receiving 75 mg m^–2^ of docetaxel, had at least one episode of neutropenic fever (16%).

Treatment discontinuation was required in 41 patients for progressive disease (26), death (1, progressive disease), AEs (9), laboratory abnormality (1), and withdrawal of consent (4). Discontinuation was treatment related in seven patients. Five patients died during follow-up, four because of progressive disease and one as a result of a cardiac event (causality unknown).

### Safety and tolerability

Treatment-emergent AEs of any causality occurring in >20% of patients receiving either the double or the triple combination are shown in Table 3. These were generally of grade 1/2 and reversible. The most frequently occurring in the doublet DLs included nausea, diarrhoea, vomiting, constipation, and fatigue. Those of grade 4 severity included neutropenia (grade 4, *n*=7; three patients experienced grade 3 neutropenia) and pulmonary embolism (grade 4, *n*=1; and one patient experienced grade 3 pulmonary embolism). Expanded safety data (>5% incidence) are available online (Supplementary Table 1).

The most frequent treatment-emergent AEs of any causality in the triplet DL were hypertension, lethargy, diarrhoea, and fatigue. Grade 4 AEs comprised febrile neutropenia (grade 4, *n*=1; one patient experienced grade 3 febrile neutropenia), hepatic encephalopathy (*n*=1), and pulmonary embolism (*n*=1).

### Efficacy

Forty-one patients were evaluable. The best response was stable disease in 21 patients (51%): 6 out of 7 at DL 1; 5 out of 21 DL 2; 3 out of 6 DL 3; and 7 out of 7 DL 4. Ten patients had stable disease lasting >6 months, with a minimum duration of 6.3 months. Tumour types associated with stable disease >6 months were prostate (*n*=3: 1 each at DLs 1, 2, and 4); sarcoma (*n*=3: 1 each at DLs 1, 3, and 4); NSCLC (*n*=2: both DL 4); hepatobiliary (*n*=1: DL 2); and squamous-cell skin carcinoma (*n*=1: DL 2). Two patients who achieved stable disease of >6 months’ duration had previously been treated with docetaxel: one with sarcoma who received the triple combination (DL 4) and one with prostate cancer who was treated with DL 1.

One patient of note, a 39-year-old male with previously treated metastatic epithelioid sarcoma, illustrated the activity of the combinations tested. At trial entry, he had disease involving the skin of the left groin, amputation stump, pubis, as well as pelvic nodes. He was entered into DL 1. After the first cycle, he noted reduced erythema and discharge from his cutaneous lesions and reduced pain. He had persisting stable radiological disease and came off study after completing the planned six courses.

**Figure 1:**
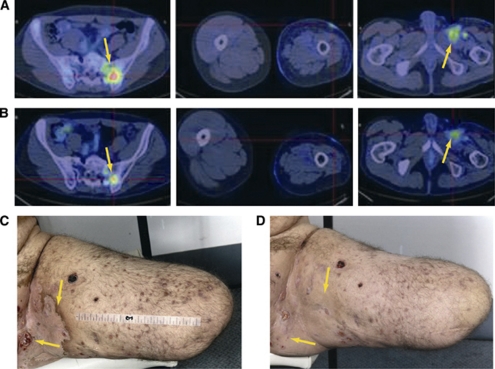
Dermatologic and metabolic responses in a 39-year-old male with metastatic epithelioid sarcoma. (**A**) Baseline PET scan. (**B**) Partial metabolic PET response following 14 days of CP-868,596 single-agent therapy. (**C**) Skin lesions prior to and (**D**) dermatologic response following 35 days of CP-868,596 single-agent therapy.

Within 4 weeks off trial, his skin lesions showed aggressive progression. Hence, he was retreated with CP-868,596 100 mg BID alone and within 2 weeks his pain again dramatically reduced. A fluordeoxyglucose positron emission tomography (FDG-PET) scan confirmed a partial metabolic response (Figure 1A and B). At 2 months, there was clinical improvement in his skin lesions (Figure 1C and D) and stable radiological disease. At 4 months, there was evidence of early progression and treatment was discontinued.

After a 4-week washout period, during which his skin lesions flared, he was re-entered into the trial into DL 4 (CP-868,596 60 mg BID/docetaxel 75 mg m^–2^/axitinib 5 mg BID). After two cycles, his skin lesions stabilised, he achieved a PET partial metabolic response, and computed tomography (CT) showed a partial response in the pelvic lymph nodes. After four courses, these nodes had completely resolved on CT with an ongoing FDG-PET response; the responses continued on restaging after six courses. He continued with CP-868,596 plus axitinib alone for a further 3 months until progressing.

### Pharmacokinetics

**Figure 2:**
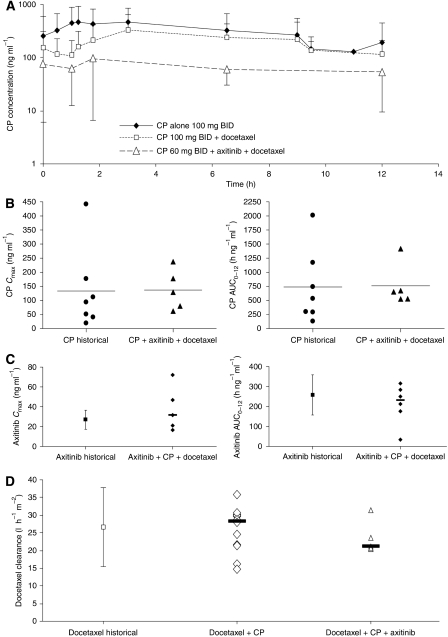
(**A**) Serum concentration profiles of CP-868,596 (CP) when administered alone or in combination with axitinib or axitinib and docetaxel. Reference data for single-agent CP-868,596 are derived from Lewis *et al* (2009). (**B**, **C**) Pharmacokinetic parameters of 60 mg CP-868,596 (CP) and axitinib when administered alone or in triple combination with docetaxel. Reference data for single-agent CP-868,596 are derived from Lewis *et al* (2009) and Pfizer data on file. Reference data for single-agent axitinib are derived from Rugo *et al* (2005). (**D**) Clearance of docetaxel when administered alone and in combination with CP-868,596 (CP) and CP-868,596 plus axitinib. Reference data for single-agent docetaxel are derived from Bruno and Sanderink (1993) and Bruno *et al* (1996).

Summary pharmacokinetic data are presented in Table 4. Key CP-868,596 pharmacokinetic parameters and plasma concentration profiles were unaffected by co-administration of docetaxel or docetaxel plus axitinib (Table 4; Figure 2A). Co-administration of docetaxel had no significant impact on CP-868,596 *C*_max_ and AUC_0–tau,_ (*P*>0.05, respectively; Table 4). Pharmacokinetic parameters of CP-868,596 were also unaffected by the co-administration of axitinib plus docetaxel relative to historical data (Figure 2B). Axitinib pharmacokinetic parameters were unaffected by co-administration of CP-868,596 or CP-868,596 plus docetaxel relative to historical data (Figure 2C). Likewise, docetaxel pharmacokinetic parameters were not affected by co-administration of CP-868,596 or CP-868,596 plus axitinib relative to historical data (Figure 2D).

### Pharmacodynamics

The DCE-MRI was performed on eight patients in DL 2 (100 mg BID CP-868,596/docetaxel 75 mg m^–2^), none of whom achieved a radiological response. There was no treatment effect using DCE-MRI in these patients as reflected by the median percentage change at day 14 relative to baseline for the following parameters: tumour volume, 14.3 (range, 0.0–33.9); vascularised volume, 16.6 (1.4–24.7); perfused AUC, −4.4% (−40.6–26.1%), and perfused *K*^trans^, 12.7% (−26.3–33.3%).

Potential biomarkers of CP-868,596 activity were analysed in 12 patients: six each in DL 1 and DL 2. At day 14, levels of pS6 in peripheral blood mononuclear cells were significantly decreased relative to baseline (*n*=11; *P*=0.021), SHP and pSHP remained unchanged (*n*=10), and levels of pAKT (*n*=12) showed a non-significant increase.

## Discussion

The concurrent modulation of TIH together with tumoural angiogenesis may be an approach to achieve adequate cytotoxic drug exposure and increase therapeutic efficacy. In order to understand the clincial effects of combining PDGFR inhibition with chemotherapy, this phase Ib trial investigated the combination of CP-868,596 and docetaxel in cancer patients. Also explored was the effect of concurrently inhibiting PDGFR and VEGFRs by adding axitinib, a potent inhibitor of VEGFR, to this combination.

The tested regimens were well tolerated and the combination of the recommended phase II dose of CP-868,596 (100 mg BID with food) (Lewis *et al*, 2009) with docetaxel (100 mg m^–2^ every 3 weeks) was feasible. However, dose escalation was limited for the triple combination because of the increased incidence of concurrent mucositis-like AEs and neutropenia relative to that expected for docetaxel alone.

The most common toxicities to date for CP-868,596 are nausea, vomiting, diarrhoea, and transaminitis (Lewis *et al*, 2009). For axitinib, the main toxicities are fatigue, nausea, diarrhoea, and hypertension (Rugo *et al*, 2005). Hence, together with the toxicities of docetaxel (Sanofi-Aventis U.S.LLC, 2008), none of the frequently observed treatment-related AEs in this study were unexpected in type. As the toxicity profiles of CP-868,596 and axitinib are largely non-overlapping, it was, therefore, assumed that they might be co-administered without additive toxicity. Unexpectedly, the incidence of concurrent mucositis-like AEs and neutropenia was elevated as noted above.

None of the patients entered in this trial achieved a major radiological response, an observation similar to that reported for the single-agent phase I study of CP-868,596 (Lewis *et al*, 2009). Nevertheless, it was unanticipated given that docetaxel is an active agent for the majority of advanced malignancy types entered into this trial (Burris *et al*, 1993; Extra *et al*, 1993; Tomiak *et al*, 1994). The lack of radiological response was also unanticipated as CP-868,596 was expected to increase the intratumoural docetaxel concentration. The effects of PDGFR inhibition on intratumoural IFP and intratumour drug concentrations were not measured in this study. However, in preclinical xenograft models, concomitant administration of PDGFR antagonists increased the tumoural uptake of paclitaxel (Pietras *et al*, 2002), and liposomal doxorubicin (Vlahovic *et al*, 2007), with increased antitumour efficacy (Vlahovic *et al*, 2007).

The lack of objective radiological response may reflect the small sample size given that the study's major end point was safety and toxicity based. It may also reflect the extent of prior chemotherapy undergone by patients entered and the heterogeneity of their disease. A recent phase II study treated patients with NSCLC (*n*=18) with second-line imatinib (a non-selective PDGFR inhibitor) plus docetaxel and reported a partial response rate of 5.5% and stable disease in 27.8% of patients (Huang *et al*, 2008). Similarly, low response rates were observed in which imatinib was combined with capecitabine in metastatic breast cancer (Chew *et al*, 2008). The selection of patients whose tumours are known to be driven by PDGFR may optimise response rates. In an *in vitro* study of PDGFR-*β* overexpressing mesothelioma cell lines, imatinib induced cytotoxicity and apoptosis, probably because of PDGFR-*β* inactivation and downstream AKT pathway inhibition (Wilmink and Richel, 2008).

The triple combination cohort was notable in that all seven evaluable patients achieved stable disease and all nine remained on therapy for a median of six cycles (range 3–16). Phase I trials of comparable doses of single-agent docetaxel reported a median of three–four cycles of administered therapy (Bissett *et al*, 1993; Tomiak *et al*, 1994). Thus, this study suggests that the combination of docetaxel with CP-868,596 and axitinib resulted in a regimen with improved disease control and tolerability. However, improved disease control may be due to the combination of axitinib with docetaxel.

Pharmacokinetic and pharmacodynamic parameters were also assessed. Pharmacokinetic data showed a lack of drug–drug interactions; indicating the feasibility of both the double and triple combinations from a pharmacokinetic perspective. Preliminary DCE-MRI data for eight patients who received CP-868,596 plus docetaxel showed no evidence of a treatment effect: this is not surprising given the lack of response observed. It may also, however, reflect the small sample size and the heterogeneity of tumour types in the study population. These findings do not corroborate the DCE-MRI changes observed on treatment with CDP860, a di-Fab anti-PGDF antibody, although the MRI parameter assessments were not identical (Jayson *et al*, 2005). In the study reported here, it is possible that tumoural CP-868,596 exposure was sub-optimal or that the MRI parameters assessed failed to detect vascular changes arising from PDGFR inhibition. Further studies are required to determine whether CP-868,596 can be combined with other anticancer drugs and whether efficacy can be improved with the use of potential biomarkers of response such as PDGFR expression.

In conclusion, combination therapy with CP-868,596 and docetaxel was well tolerated and no unfavourable drug–drug interactions were shown. The expected additive clinical efficacy was not observed and may not support the clinical development of this combination in non-selected patient populations. However, the addition of axitinib to the combination resulted in greater-than-expected benefits in terms of prolonged stable disease and tolerability. The separate inhibition of individual receptor targets in this study resulted in unexpected findings, suggesting that this approach may be worthy of further study.

## Figures and Tables

**Table 1:** Baseline demographics

	**CP-868,596 plus docetaxel (*n*=39)**	**CP-868,596 plus docetaxel plus axitinib (*n*=9)**	**Overall (*n*=48)**
Age in years, median (range)	55.0 (25–78)	59.0 (31–74)	57 (25–78)
			
*Gender, n* (%)			
Male	28 (71.8)	9 (100)	37 (77.1)
Female	11 (28.2)	0	11 (22.9)
			
*ECOG performance status, n* (%)			
0	22 (56.4)	1 (11.1)	23 (47.9)
1	16 (41.0)	7 (77.8)	23 (47.9)
2	1 (2.6)	1 (11.1)	2 (4.2)
			
*Primary diagnosis*, n			
NSCLC	7	3	10
Prostate cancer	5	1	6
Oesophageal carcinoma	4	1	5
Ewing's sarcoma	3	0	3
Sarcoma	5	0	5
Small cell lung cancer	3	0	2
Colorectal cancer	3	0	3
Other	9	4	14
			
*Prior cancer therapy*			
Radiotherapy	27 (69.2)	5 (55.6)	32 (66.7)
Surgery	24 (61.5)	5 (55.6)	29 (60.4)
Chemotherapy	38 (97.4)	9 (100)	47 (97.9)
1 prior regimen	16 (41.0)	5 (55.6)	21 (43.8)
2 prior regimens	9 (23.1)	3 (33.3)	12 (25.0)
⩾3 prior regimens	13 (33.3)	1 (11.1)	14 (29.2)
Hormonal therapy	7 (17.9)	1 (11.1)	8 (16.7)
Other	4 (10.3)	1 (11.1)	5 (10.4)

Abbreviations: ECOG=Eastern Cooperative Oncology Group; NSCLC=non-small cell lung cancer.

**Table 2:** Dosing and DLT summary by patient cohort

**Dose level**	**CP-856,596**	**Docetaxel**	**Axitinib**	**Patients evaluable for DLT** **in the dose-escalation cohorts**^a^	**Total number of patients**	**DLT (*n*)**	**Median duration of treatment, months (range)**	**Median number of cycles administered (range)**	**Number of patients with dose reduction**
1	60 mg BID	75 mg m^–2^	0	6	7	Febrile neutropenia (1)	3.45 (0.23–9.21)	4.0 (1.0–11.0)	0
2	100 mg BID	75 mg m^–2^	0	6	25	Febrile neutropenia (1)^b^ Nausea/vomiting (1)^b^	1.48 (0.23–8.36)	2.0 (1.0–11.0)	0
3	100 mg BID	100 mg m^–2^	0	6	7	None	2.07 (0.69–4.84)	2.0 (1.0–6.0)	0
4	60 mg BID	75 mg m^–2^	5 mg BID	9	9	*Klebsiella pneumonae* infection associated with febrile neutropenia (1) Febrile neutropenia (1)	5.3 (1.97–12.47)	6.0 (3.0–16.0)	1

Abbreviations: DLT=dose-limiting toxicity; BID=twice daily.

a Patients evaluable for DLT either discontinued the study during cycle 1 because of toxicity or completed cycle 1.

b DLT observed in patients enrolled into the expansion cohort.

**Table 3:** Treatment-emergent (all-causality) AEs of interest experienced by ⩾20% of the study population in either treatment group (all cycles)^a^

	**CP-856,596+docetaxel (*n*=39)**			**CP-856,596+docetaxel+axitinib (*n*=9)**		
**AE**	**Grade 1/2, *n* (%)**	**Grade 3/4, *n* (%)**	**Total**	**Grade 1/2, *n* (%)**	**Grade 3/4, *n* (%)**	**Total**
Nausea	27 (69.2)	5 (12.8)	32 (82.1)	4 (44.4)	0	4 (44.4)
Diarrhoea	22 (56.4)	3 (7.7)	25 (64.1)	5 (55.6)	0	5 (55.6)
Vomiting	22 (56.4)	1 (2.6)	23 (59.0)	4 (44.4)	0	4 (44.4)
Constipation	20 (51.3)	0	20 (51.3)	4 (44.4)	0	4 (44.4)
Fatigue	11 (28.2)	6 (15.4)	17 (43.6)	4 (44.4)	1 (11.1)	5 (55.6)
Anaemia	10 (25.6)	6^b^ (15.4)	16 (41.0)	1 (11.1)	0	1 (11.1)
Dyspnoea	10 (25.6)	4 (10.3)	14 (35.9)	4 (44.4)	0	4 (44.4)
Lethargy	10 (25.6)	3 (7.7)	13 (33.3)	5 (55.6)	1 (11.1)	6 (66.7)
Neutropenia	3 (7.7)	10^c^ (25.6)	13 (33.3)	0	0	0
Pyrexia	11 (28.2)	2 (5.1)	13 (33.3)	4 (44.4)	0	4 (44.4)
Anorexia	11 (28.2)	1 (2.6)	12 (30.8)	2 (22.2)	0	2 (22.2)
Rash^d^	12 (30.8)	0	12 (30.8)	3 (33.3)	0	3 (33.3)
Peripheral oedema	11 (28.2)	0	11 (28.2)	0	0	0
Mucosal inflammation	8 (20.5)	1 (2.6)	9 (23.1)	2 (22.2)	2 (22.2)	4 (44.4)
Dysguesia	9 (23.1)	0	9 (23.1)	2 (22.2)	0	2 (22.2)
Pain	6 (15.3)	3 (7.7)	9 (23.1)	I (11.1)	0	1 (11.1)
Headache	8 (20.5)	0	8 (20.5)	I (11.1)	0	1 (11.1)
Cough	6 (15.4)	0	6 (15.4)	2 (22.2)	0	2 (22.2)
Tachycardia	8 (20.5)	0	8 (20.5)	1 (11.1)	0	1 (11.1)
Febrile neutropenia	1 (2.6)	4 (10.3)	5 (12.8)	0	2^b^ (22.2)	2 (22.2)
Dehydration	5 (12.8)	0	5 (12.8)	1 (11.1)	1 (11.1)	2 (22.2)
ALT increased	4 (10.3)	0	4 (10.3)	2 (22.2)	0	2 (22.2)
Hypertension	3 (7.7)	0	3 (7.7)	6 (66.7)	0	6 (66.7)
Epistaxis	3 (7.7)	0	3 (7.7)	2 (22.2)	0	2 (22.2)
Peripheral neuropathy	1 (2.6)	0	1 (2.6)	1 (11.1)	1 (11.1)	2 (22.2)
Dysphonia	1 (2.6)	0	1 (2.6)	4 (44.4)	0	4 (44.4)
Lacrimation increased	0	0	0	3 (33.3)	0	3 (33.3)
Abdominal discomfort	1 (2.6)	0	1 (2.6)	2 (22.0)	0	2 (22.0)
Chest pain	3 (7.7)	0	3 (7.7)	2 (22.2)	0	2 (22.2)
Hypotension	3 (7.7)	0	3 (7.7)	1 (11.1)	1 (11.1)	2 (22.2)
Insomnia	4 (10.3)	0	4 (10.3)	2 (22.2)	0	2 (22.2)
PPE	1 (2.6)	0	1 (2.6)	2 (22.2)	0	2 (22.2)
Paraesthesia	3 (7.7)	0	3 (7.7)	2 (22.2)	0	2 (22.2)
Toothache	0	0	0	2 (22.2)	0	2 (22.2)

Abbreviations: AE=adverse event; ALT=alanine amino transferase; PPE=palmar–plantar erythrodysaesthia syndrome.

a The safety population comprised all patients who received at least one dose of study medication.

b One grade 4.

c Seven grade 4.

d Pooled data: rash, rash erythematous, rash generalised, rash maculopapular, rash pruritic, and heat rash.

**Table 4:** CP-868,596 pharmacokinetic summary

	**Pharmacokinetic parameters**		
**Regimens**	***T*_max_ (h)**	***C*_max_ (ng ml^–1^)** a	**AUC_(0–12)_ (h ng ml^–1^)** b
*CP-868,596 (100 mg BID fed)*			
*N*	13	13	13
Mean	2.30	493	3200
Median	1.75	419	2240
CV%	64	86	96
			
*CP-868,596 (100 mg BID fed)*+*docetaxel (75 mg m*^*–2*^*)*			
*N*	13	13	13
Mean	2.98	360	2540
Median	3	326	1640
CV%	44	83	89
			
*CP-868,596 (60 mg BID fed)*+*axitinib (5 mg BID fed)*+*docetaxel (75 mg m*^*–2*^*)*			
*N*	5	5	5
Mean	4.4	137	760
Median	1.75	129	654
CV%	111	52	49

Abbreviations: AUC=area under the curve; BID=twice daily.

CV%=100% × (s.d./mean).

a *C*_max_: CP-868,596 *vs* CP-868,596 plus docetaxel: *P*=0.055.

b AUC_tau (0–12 h)_ CP-868,596 *vs* CP-868,596 plus docetaxel: *P*=0.117.
