# Supplementary material for: Phase Ib study of CP-868,596, a PDGFR inhibitor, combined with docetaxel with or without axitinib, a VEGFR inhibitor
Source: Br J Cancer. 2010 Oct 19;103(10):1554–61. doi: 10.1038/sj.bjc.6605941 (PMC2990584; doi:10.1038/sj.bjc.6605941)
Supplement: Supplementary Information [file 6605941x1.doc]

**Supplementary Figure 1. Study Design.**

**
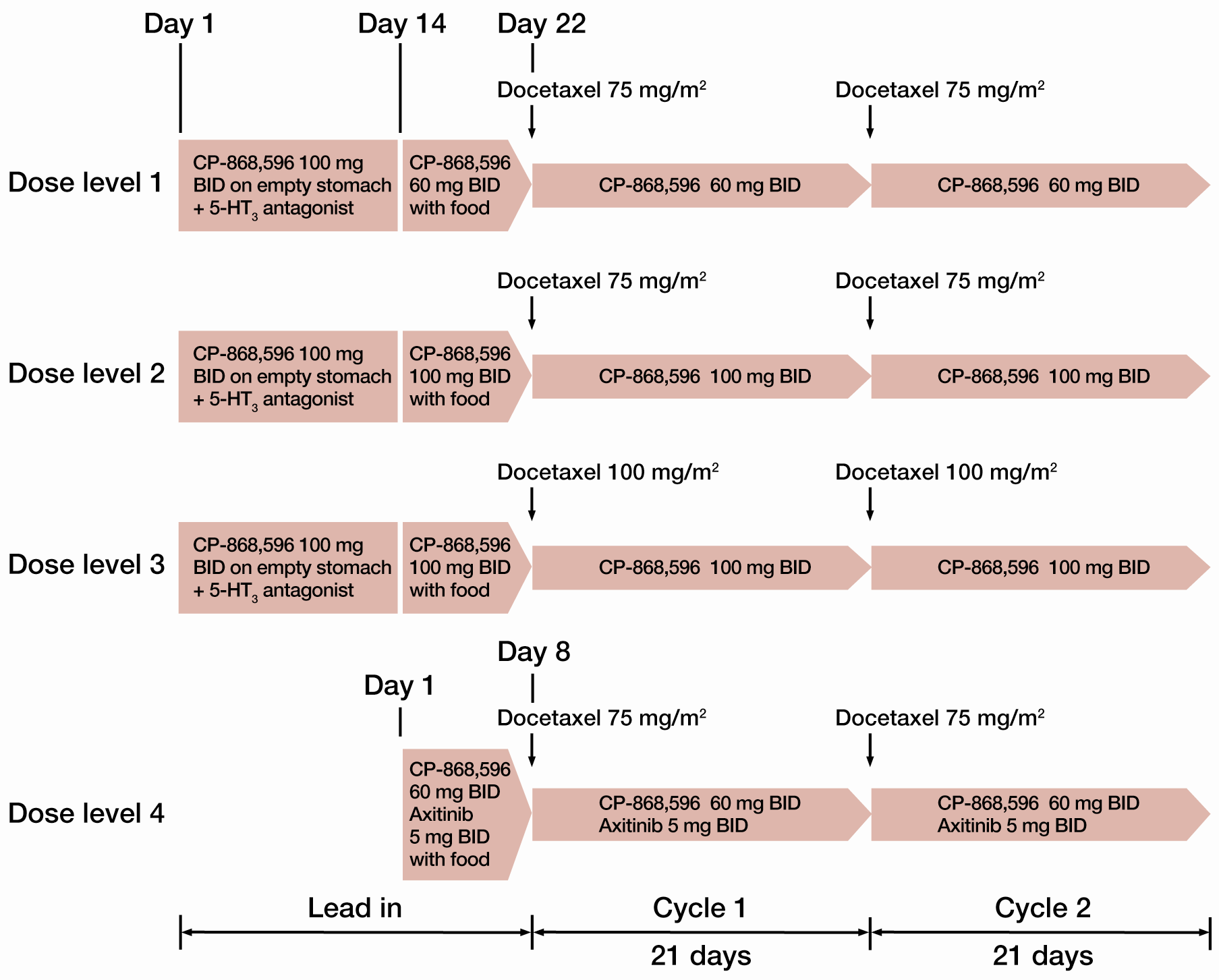
**Dose escalations and de-escalations planned but not investigated comprised dose levels –1, 4a, and 4b. Dose level –1 consisted of CP-868,596 40 mg BID plus docetaxel 75 mg/m^2^ and was to be administered only if required secondary to toxicity at the starting dose level, level 1 (CP-868,596 60 mg BID plus docetaxel 75 mg/m^2^). Dose level 4a (CP-868,596 40 mg BID, docetaxel 75 mg/m^2^, and axitinib 5 mg BID) was to be used only if required secondary to toxicity in the starting dose level, level 4 (CP-868,596 60 mg BID plus docetaxel 75 mg/m^2^ plus axitinib 5 mg BID). Dose level 4b, CP-868,596 100 mg BID plus docetaxel 75 mg/m^2^ plus axitinib 5 mg BID, was rescinded as a result of observed toxicity at dose level 4.

**Supplemantary Table 1.** Treatment-emergent (all-causality) adverse events of interest experienced by ≥5% of the study population in either treatment group (all cycles)

| **Adverse event** | **CP-856,596 + docetaxel**  **(*n* = 39)** | | | **CP-856,596 + docetaxel + axitinib (*n* = 9)** | | |
| --- | --- | --- | --- | --- | --- | --- |
|  | **Grade 1/2, n (%)** | **Grade 3/4, n (%)** | **Total, n (%)** | **Grade 1/2, n (%)** | **Grade 3/4, n (%)** | **Total, n (%)** |
| Nausea | 27 (69.2) | 5 (12.8) | 32 (82.1) | 4 (44.4) | 0 | 4 (44.4) |
| Diarrhoea | 22 (56.4) | 3 (7.7) | 25 (64.1) | 5 (55.6) | 0 | 5 (55.6) |
| Vomiting | 22 (56.4) | 1 (2.6) | 23 (59.0) | 4 (44.4) | 0 | 4 (44.4) |
| Constipation | 20 (51.3) | 0 | 20 (51.3) | 4 (44.4) | 0 | 4 (44.4) |
| Fatigue | 11 (28.2) | 6 (15.4) | 17 (43.6) | 4 (44.4) | 1 (11.1) | 5 (55.6) |
| Anaemia | 10 (25.6) | 6 (15.4) | 16 (41.0) | 1 (11.1) | 0 | 1 (11.1) |
| Dyspnoea | 10 (25.6) | 4 (10.3) | 14 (35.9) | 4 (44.4) | 0 | 4 (44.4) |
| Lethargy | 10 (25.6) | 3 (7.7) | 13 (33.3) | 5 (55.6) | 1 (11.1) | 6 (66.7) |
| Neutropenia | 3 (7.7) | 10 (25.6) | 13 (33.3) | 0 | 0 | 0 |
| Pyrexia | 11 (28.2) | 2 (5.1) | 13 (33.3) | 4 (44.4) | 0 | 4 (44.4) |
| Anorexia | 11 (28.2) | 1 (2.6) | 12 (30.8) | 2 (22.2) | 0 | 2 (22.2) |
| Oedema peripheral | 11 (28.2) | 0 | 11 (28.2) | 0 | 0 | 0 |
| Alopecia | 9 (23.1) | 0 | 9 (23.1) | 1 (11.1) | 0 | 1 (11.1) |
| Dysgeusia | 9 (23.1) | 0 | 9 (23.1) | 2 (22.2) | 0 | 2 (22.2) |
| Mucosal inflammation | 8 (20.5) | 1 (2.6) | 9 (23.1) | 2 (22.2) | 2 (22.2) | 4 (44.4) |
| Pain | 6 (15.4) | 3 (7.7) | 9 (23.1) | 1 (11.1) | 0 | 1 (11.1) |
| Headache | 8 (20.5) | 0 | 8 (20.5) | 1 (11.1) | 0 | 1 (11.1) |
| Tachycardia | 8 (20.5) | 0 | 8 (20.5) | 1 (11.1) | 0 | 1 (11.1) |
| Gamma-glutamyltransferase increased | 6 (15.4) | 1 (2.6) | 7 (17.9) | 0 | 0 | 0 |
| Hyperglycaemia | 5 (12.8) | 2 (5.1) | 7 (17.9) | 0 | 0 | 0 |
| Abdominal pain | 5 (12.8) | 1 (2.6) | 6 (15.4) | 1 (11.1) | 0 | 1 (11.1) |
| Blood alkaline phosphatase increased | 5 (12.8) | 1 (2.6) | 6 (15.4) | 0 | 0 | 0 |
| Cough | 6 (15.4) | 0 | 6 (15.4) | 2 (22.2) | 0 | 2 (22.2) |
| Dizziness | 6 (15.4) | 0 | 6 (15.4) | 0 | 0 | 0 |
| Aspartate aminotransferase increased | 5 (12.8) | 0 | 5 (12.8) | 1 (11.1) | 0 | 1 (11.1) |
| Back pain | 4 (10.3) | 1 (2.6) | 5 (12.8) | 0 | 0 | 0 |
| Decreased appetite | 5 (12.8) | 0 | 5 (12.8) | 1 (11.1) | 0 | 1 (11.1) |
| Dehydration | 5 (12.8) | 0 | 5 (12.8) | 1 (11.1) | 1 (11.1) | 2 (22.2) |
| Dry skin | 5 (12.8) | 0 | 5 (12.8) | 1 (11.1) | 0 | 1 (11.1) |
| Febrile neutropenia | 1 (2.6) | 4 (10.3) | 5 (12.8) | 0 | 2 (22.2) | 2 (22.2) |
| Hypoalbuminaemia | 5 (12.8) | 0 | 5 (12.8) | 0 | 0 | 0 |
| Somnolence | 5 (12.8) | 0 | 5 (12.8) | 1 (11.1) | 0 | 1 (11.1) |
| Stomatitis | 4 (10.3) | 1 (2.6) | 5 (12.8) | 0 | 0 | 0 |
| Alanine aminotransferase increased | 4 (10.3) | 0 | 4 (10.3) | 2 (22.2) | 0 | 2 (22.2) |
| Arthralgia | 4 (10.3) | 0 | 4 (10.3) | 0 | 0 | 0 |
| Blood albumin decreased | 4 (10.3) | 0 | 4 (10.3) | 0 | 0 | 0 |
| Dyspepsia | 4 (10.3) | 0 | 4 (10.3) | 0 | 0 | 0 |
| Hyponatraemia | 3 (7.7) | 1 (2.6) | 4 (10.3) | 0 | 0 | 0 |
| Insomnia | 4 (10.3) | 0 | 4 (10.3) | 2 (22.2) | 0 | 2 (22.2) |
| Myalgia | 4 (10.3) | 0 | 4 (10.3) | 1 (11.1) | 0 | 1 (11.1) |
| White blood cell count decreased | 1 (2.6) | 3 (7.7) | 4 (10.3) | 0 | 0 | 0 |
| Chest pain | 3 (7.7) | 0 | 3 (7.7) | 2 (22.2) | 0 | 2 (22.2) |
| Chills | 3 (7.7) | 0 | 3 (7.7) | 1 (11.1) | 0 | 1 (11.1) |
| Confusional state | 3 (7.7) | 0 | 3 (7.7) | 1 (11.1) | 0 | 1 (11.1) |
| Epistaxis | 3 (7.7) | 0 | 3 (7.7) | 2 (22.2) | 0 | 2 (22.2) |
| Gamma-glutamyltransferase | 2 (5.1) | 1 (2.6) | 3 (7.7) | 0 | 0 | 0 |
| Haemoglobin decreased | 3 (7.7) | 0 | 3 (7.7) | 0 | 0 | 0 |
| Hyperkalaemia | 3 (7.7) | 0 | 3 (7.7) | 0 | 0 | 0 |
| Hypermagnesaemia | 3 (7.7) | 0 | 3 (7.7) | 0 | 0 | 0 |
| Hypertension | 3 (7.7) | 0 | 3 (7.7) | 6 (66.7) | 0 | 6 (66.7) |
| Hypocalcaemia | 2 (5.1) | 1 (2.6) | 3 (7.7) | 0 | 0 | 0 |
| Hypotension | 3 (7.7) | 0 | 3 (7.7) | 1 (11.1) | 1 (11.1) | 2 (22.2) |
| Hypoxia | 1 (2.6) | 2 (5.1) | 3 (7.7) | 0 | 0 | 0 |
| Musculoskeletal pain | 3 (7.7) | 0 | 3 (7.7) | 1 (11.1) | 0 | 1 (11.1) |
| Neuropathy peripheral | 1 (2.6) | 2 (5.1) | 3 (7.7) | 1 (11.1) | 1 (11.1) | 2 (22.2) |
| Non-cardiac chest pain | 3 (7.7) | 0 | 3 (7.7) | 0 | 0 | 0 |
| Oral candidiasis | 3 (7.7) | 0 | 3 (7.7) | 1 (11.1) | 0 | 1 (11.1) |
| Paraesthesia | 3 (7.7) | 0 | 3 (7.7) | 2 (22.2) | 0 | 2 (22.2) |
| Productive cough | 2 (5.1) | 1 (2.6) | 3 (7.7) | 1 (11.1) | 0 | 1 (11.1) |
| Rash | 3 (7.7) | 0 | 3 (7.7) | 2 (22.2) | 0 | 2 (22.2) |
| Rash erythematous | 3 (7.7) | 0 | 3 (7.7) | 2 (22.2) | 0 | 2 (22.2) |
| Abdominal distension | 2 (5.1) | 0 | 2 (5.1) | 0 | 1 (11.1) | 1 (11.1) |
| Abdominal pain upper | 2 (5.1) | 0 | 2 (5.1) | 0 | 0 | 0 |
| Asthenia | 2 (5.1) | 0 | 2 (5.1) | 0 | 0 | 0 |
| Atrial fibrillation | 2 (5.1) | 0 | 2 (5.1) | 0 | 0 | 0 |
| Blood calcium decreased | 2 (5.1) | 0 | 2 (5.1) | 0 | 0 | 0 |
| Blood magnesium increased | 2 (5.1) | 0 | 2 (5.1) | 0 | 0 | 0 |
| Cellulitis | 1 (2.6) | 1 (2.6) | 2 (5.1) | 0 | 0 | 0 |
| Claustrophobia | 2 (5.1) | 0 | 2 (5.1) | 0 | 0 | 0 |
| Depression | 1 (2.6) | 1 (2.6) | 2 (5.1) | 1 (11.1) | 0 | 1 (11.1) |
| Disease progression | 0 | 1 (2.6) | 2 (5.1) | 0 | 0 | 0 |
| Disorientation | 2 (5.1) | 0 | 2 (5.1) | 0 | 0 | 0 |
| Dysphagia | 2 (5.1) | 0 | 2 (5.1) | 0 | 0 | 0 |
| Dysuria | 2 (5.1) | 0 | 2 (5.1) | 0 | 0 | 0 |
| Fall | 2 (5.1) | 0 | 2 (5.1) | 0 | 0 | 0 |
| Fluid retention | 2 (5.1) | 0 | 2 (5.1) | 0 | 0 | 0 |
| Haemoptysis | 2 (5.1) | 0 | 2 (5.1) | 0 | 0 | 0 |
| Haemorrhoids | 2 (5.1) | 0 | 2 (5.1) | 0 | 0 | 0 |
| Herpes zoster | 2 (5.1) | 0 | 2 (5.1) | 0 | 0 | 0 |
| Impaired healing | 2 (5.1) | 0 | 2 (5.1) | 0 | 0 | 0 |
| Liver function test abnormal | 2 (5.1) | 0 | 2 (5.1) | 0 | 0 | 0 |
| Muscle spasms | 2 (5.1) | 0 | 2 (5.1) | 0 | 0 | 0 |
| Muscular weakness | 0 (0.0) | 2 (5.1) | 2 (5.1) | 0 | 0 | 0 |
| Musculoskeletal chest pain | 2 (5.1) | 0 | 2 (5.1) | 0 | 0 | 0 |
| Nail disorder | 2 (5.1) | 0 | 2 (5.1) | 1 (11.1) | 0 | 1 (11.1) |
| Oedema | 2 (5.1) | 0 | 2 (5.1) | 0 | 0 | 0 |
| Pain in extremity | 2 (5.1) | 0 | 2 (5.1) | 0 | 0 | 0 |
| Pleural effusion | 2 (5.1) | 0 | 2 (5.1) | 1 (11.1) | 0 | 1 (11.1) |
| Protein total decreased | 2 (5.1) | 0 | 2 (5.1) | 0 | 0 | 0 |
| Pulmonary embolism | 0 (0.0) | 2 (5.1) | 2 (5.1) | 0 | 1 (11.1) | 1 (11.1) |
| Pulmonary oedema | 1 (2.6) | 1 (2.6) | 2 (5.1) | 0 | 0 | 0 |
| Sinus congestion | 2 (5.1) | 0 | 2 (5.1) | 0 | 0 | 0 |
| Skin exfoliation | 2 (5.1) | 0 | 2 (5.1) | 0 | 0 | 0 |
| Thrombocytopenia | 2 (5.1) | 0 | 2 (5.1) | 0 | 0 | 0 |
| Urinary tract infection | 2 (5.1) | 0 | 2 (5.1) | 0 | 0 | 0 |
| Visual disturbance | 2 (5.1) | 0 | 2 (5.1) | 0 | 0 | 0 |
| Weight decreased | 2 (5.1) | 0 | 2 (5.1) | 0 | 0 | 0 |
| Dysphonia | 1 (2.6) | 0 | 1 (2.6) | 4 (44.4) | 0 | 4 (44.4) |
| Abdominal discomfort | 1 (2.6) | 0 | 1 (2.6) | 2 (22.2) | 0 | 2 (22.2) |
| Palmar-plantar erythrodysaesthesia syndrome | 1 (2.6) | 0 | 1 (2.6) | 2 (22.2) | 0 | 2 (22.2) |
| Blood bilirubin increased | 1 (2.6) | 0 | 1 (2.6) | 1 (11.1) | 0 | 1 (11.1) |
| Blood magnesium decreased | 1 (2.6) | 0 | 1 (2.6) | 1 (11.1) | 0 | 1 (11.1) |
| Groin pain | 1 (2.6) | 0 | 1 (2.6) | 1 (11.1) | 0 | 1 (11.1) |
| Onychoclasis | 1 (2.6) | 0 | 1 (2.6) | 1 (11.1) | 0 | 1 (11.1) |
| Oral pain | 1 (2.6) | 0 | 1 (2.6) | 1 (11.1) | 0 | 1 (11.1) |
| Peripheral sensory neuropathy | 1 (2.6) | 0 | 1 (2.6) | 1 (11.1) | 0 | 1 (11.1) |
| Pneumonia | 0 | 1 (2.6) | 1 (2.6) | 0 | 1 (11.1) | 1 (11.1) |
| Pruritus | 1 (2.6) | 0 | 1 (2.6) | 0 | 0 | 0 |
| Tremor | 0 | 1 (2.6) | 1 (2.6) | 1 (11.1) | 0 | 1 (11.1) |
| Lacrimation increased | 0 | 0 | 0 | 3 (33.3) | 0 | 3 (33.3) |
| Toothache | 0 | 0 | 0 | 2 (22.2) | 0 | 2 (22.2) |
| Ageusia | 0 | 0 | 0 | 1 (11.1) | 0 | 1 (11.1) |
| Agitation | 0 | 0 | 0 | 1 (11.1) | 0 | 1 (11.1) |
| Apathy | 0 | 0 | 0 | 1 (11.1) | 0 | 1 (11.1) |
| Bone pain | 0 | 0 | 0 | 1 (11.1) | 0 | 1 (11.1) |
| Dermatitis acneiform | 0 | 0 | 0 | 1 (11.1) | 0 | 1 (11.1) |
| Extravasation | 0 | 0 | 0 | 1 (11.1) | 0 | 1 (11.1) |
| Flank pain | 0 | 0 | 0 | 1 (11.1) | 0 | 1 (11.1) |
| Folliculitis | 0 | 0 | 0 | 1 (11.1) | 0 | 1 (11.1) |
| Glossodynia | 0 | 0 | 0 | 1 (11.1) | 0 | 1 (11.1) |
| Hallucination | 0 | 0 | 0 | 1 (11.1) | 0 | 1 (11.1) |
| Hepatic encephalopathy | 0 | 0 | 0 | 0 | 1 (11.1) | 1 (11.1) |
| Hypoaesthesia | 0 | 0 | 0 | 1 (11.1) | 0 | 1 (11.1) |
| Infection | 0 | 0 | 0 | 0 | 1 (11.1) | 1 (11.1) |
| Klebsiella infection | 0 | 0 | 0 | 0 | 1 (11.1) | 1 (11.1) |
| Lip dry | 0 | 0 | 0 | 1 (11.1) | 0 | 1 (11.1) |
| Melaena | 0 | 0 | 0 | 1 (11.1) | 0 | 1 (11.1) |
| Nasal congestion | 0 | 0 | 0 | 1 (11.1) | 0 | 1 (11.1) |
| Night sweats | 0 | 0 | 0 | 1 (11.1) | 0 | 1 (11.1) |
| Oral intake reduced | 0 | 0 | 0 | 1 (11.1) | 0 | 1 (11.1) |
| Pancreatitis | 0 | 0 | 0 | 1 (11.1) | 0 | 1 (11.1) |
| Paraesthesia oral | 0 | 0 | 0 | 1 (11.1) | 0 | 1 (11.1) |
| Periodontal destruction | 0 | 0 | 0 | 1 (11.1) | 0 | 1 (11.1) |
| Pleuritic pain | 0 | 0 | 0 | 1 (11.1) | 0 | 1 (11.1) |
| Psychomotor hyperactivity | 0 | 0 | 0 | 1 (11.1) | 0 | 1 (11.1) |
| Rhinorrhoea | 0 | 0 | 0 | 1 (11.1) | 0 | 1 (11.1) |
| Sinusitis | 0 | 0 | 0 | 1 (11.1) | 0 | 1 (11.1) |
| Skin discolouration | 0 | 0 | 0 | 1 (11.1) | 0 | 1 (11.1) |
| Skin laceration | 0 | 0 | 0 | 1 (11.1) | 0 | 1 (11.1) |
| Skin ulcer | 0 | 0 | 0 | 1 (11.1) | 0 | 1 (11.1) |
| Tooth abscess | 0 | 0 | 0 | 1 (11.1) | 0 | 1 (11.1) |
| Upper respiratory tract infection | 0 | 0 | 0 | 0 | 1 (11.1) | 1 (11.1) |
| Urinary retention | 0 | 0 | 0 | 0 | 1 (11.1) | 1 (11.1) |
